# Stress Enhances Proinflammatory Platelet Activity: the Impact of Acute and Chronic Mental Stress

**DOI:** 10.1007/s11481-020-09945-4

**Published:** 2020-08-05

**Authors:** Pia Koudouovoh-Tripp, Katharina Hüfner, Jonas Egeter, Christina Kandler, Johannes M. Giesinger, Sieghart Sopper, Christian Humpel, Barbara Sperner-Unterweger

**Affiliations:** 1 Department of Psychiatry, Psychotherapy and Psychosomatics, Division of Psychiatry I, Medical University Innsbruck, Innsbruck, Austria; 2 Department of Psychiatry, Psychotherapy and Psychosomatics, Division of Psychiatry II, Medical University Innsbruck, Innsbruck, Austria; 3 Clinic for Hematology and Oncology, Flow Cytometry Unit, Medical University Innsbruck, Innsbruck, Austria; 4 Laboratory of Psychiatry and Exp. Alzheimer’s Research, Medical University Innsbruck, Innsbruck, Austria

**Keywords:** Acute mental stress, Chronic mental stress, Platelets, Bioactivity, Aggregability, CD62P, CD63, Platelet leucocyte aggregates

## Abstract

The role of platelets in hemostasis and thrombosis has long been recognized, recently their contribution to immunological and inflammatory processes is emerging. Platelets could be the missing link between cardiovascular disease, chronic stress and depressive symptoms. Both physical and mental stressors cause platelet activation reflected by changes in platelet bioactivity and aggregation. Here we evaluate the proinflammatory platelet response to acute and chronic mental stress. In a prospective study design an acute mental stress test was administered to 55 healthy male participants once without and once in the presence of chronic mental stress. Blood was collected prior to and at three time points following an acute mental stress test (0, 30, 60 min). Platelet proinflammatory activation markers, were assessed using FACS analysis and aggregability was measured in response to ADP or epinephrine using PFA-100. A linear mixed model was used for analysis. Chronic mental stress lead to a significant increase in state anxiety (*p* < 0.001), depressive symptoms (*p* = 0.045) and perceived stress (*p* = 0.001). The factor “chronic mental stress” was significantly associated with increased numbers of CD63+ platelets (*p* = 0.009). The factor “acute mental stress” was associated with alterations in CD62P+ platelets (*p* < 0.001), CD63+ platelets (*p* = 0.011), PAC-1+ platelets (p < 0.001) as well as platelet leucocyte aggregates (*p* = 0.019). The recovery of CD62P function following the acute mental stress exposure was significantly impaired by chronic stress (*p* = 0.023). Aggregation was affected by chronic and acute mental stress. In conclusion, mental stress is linked to an increased and prolonged proinflammatory platelet bioactivity. This proinflammatory and immunomodulatory stimuli could help to explain the link between mental and somatic disorders.

**Figure:**
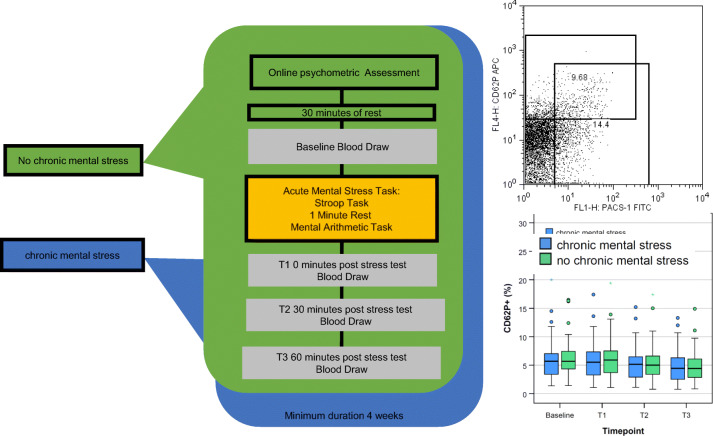
Graphical Abstract

Graphical Abstract

## Introduction

Platelets are the smallest cell fragments in the human body, (Jurk and Kehrel 2005). Primarily known for their effector role in hemostasis, thrombosis and wound healing, recent investigations have highlighted their engagement in the immune system. Platelets can be regarded as effector cells in the innate immune system. Furthermore, they are able to cross talk with the adaptive immune system by secreting pro-inflammatory and immunomodulatory proteins (Semple et al. 2011). These molecules are stored in three types of storage granules: dense granules, alpha granules, and lysosomes. Each granule represents a specific set of pro-inflammatory and immunomodulatory compounds. The dense granules mainly store serotonin, adenosine diphosphate (ADP) and CD63 (Jurk and Kehrel 2005) while the alpha granules store adhesion molecules and immune modulatory molecules like P-Selectin (CD62P), beta-Thromboglobulin, Platelet factor 4 and CD40.

Activated through different stressors including pathogens, tissue damage, physical activity or mental stress, platelets perform a rapid shape change which leads to presentation of surface markers such as CD62P and CD63 (Israels et al. 2001) and secretion of over 300 bioactive compounds into the cellular microenvironment (Semple et al. 2011). Activation of platelets also leads to the release of microvesicles and exosomes into the circulation which contain many markers of activation (Zaldivia et al. 2017). Platelets are a popular model in psychiatric disorders because they display serotonergic parameters similar to those in the central nervous system e.g. serotonin receptors as well as the serotonin transporter and the fact that they store over 80% of the peripheral serotonin (Stahl 1985; Muck-Seler et al. 2004; Zhuang et al. 2018). Since platelets also have an important role in the immune system, they may serve as an easily accessible tool to investigate mental stress induced proinflammatory states.

Changes in platelet function could also be the missing link to explain the statistical association of cardiovascular disease, such as myocardial infarction and stroke with mental health (Frasure-Smith and Lespérance 2010). The platelet activation marker P-Selectin, for example, plays numerous important and differential roles, such as in the formation of leukocyte-platelet aggregates, by promoting interactions between platelets and the vascular endothelium, as well as in thrombus formation during hemostasis (Imhof and Dunon 1995). Moreover, P-Selectin may predict vascular damage associated with hypertension and acute coronary syndromes (Itoh et al. 1995) or cardiac mortality in patients with atrial fibrillation (Heeringa et al. 2006). Platelet-leukocyte interactions stimulate the release of pro-inflammatory and pro-thrombotic factors which promote atherosclerosis (Imhof and Dunon 1995). More recently the role of platelet derived microvesicles and exosomes (Tao et al. 2017) in a variety of pathological processes and diseases including inflammation, atherosclerosis or acute coronary syndrome has gained attention (Zaldivia et al. 2017).

The term “stress” describes environmental stimuli, which threaten an organism’s hemostasis. The stress response system is mainly represented through the hypothalamus pituary axis and the sympathoadrenal system. The human body’s reactions to stress are one of the most fundamental adaptive resources. Chronic stress can lead to sustained alterations like a dysregulated hypothalamic–pituitary–adrenal (HPA) axis (Chrousos 2009; Agorastos et al. 2018) a chronic proinflammatory state (Dhabhar 2014) and subsequent disruption of neurotransmitter pathways. These deleterious aspects of the stress reaction can be termed allostatic load (McEwen 2005).

Regarding the impact of stress, the role of perceived stress and coping is of great importance. Chronic stress is thought to sensitize the organism towards upcoming acute stressors and to kindle for the onset of major depressive disorders (Monroe and Harkness 2005). A history of childhood abuse (a form of chronic stress) leads to a an increased Interleukin 6 (IL-6) in response to acute daily stressors compared to individuals without a history of chronic stress (Gouin et al. 2012). Chronic stress, in the form of caring for a relative with dementia, was found to impair platelet CD62P recovery (Aschbacher et al. 2008) and subclinical depressive symptoms were associated with significantly increased platelet CD62P expression following an acute stress test in individuals with chronic stress (dementia caregivers) (Aschbacher et al. 2009a).

The aim of our study was to evaluate the effect of chronic and acute mental stress as well as their interaction on platelet aggregation and immunomodulatory function. The proinflammatory platelet marker set consisted of platelet surface P-selectin (CD62P), the dense granule and lysosomal marker CD63, PAC-1 (activated GP IIb/IIIa), and platelet leucocyte aggregates (CD45high42b+, PLA).

## Methods

### Ethics Statement

Our study was approved by the ethics committee of the Innsbruck Medical University, Austria. Informed written consent was obtained from all participants prior to inclusion in the study. The authors report no conflicts of interest.

### Participants

Participants were recruited through our university program. We included somatically and mentally healthy medical students. To avoid interference with hormonal cycle only male medical students were included. Past medical history was obtained through anamnestic questionnaire. The following inclusion criteria were applied: male medical students, no mental disorder, no somatic disorder requiring medical attention, non-smokers, no adherence to any specific diet, no caffeinated or alcoholic beverages prior to the assessment, no excessive exercise or sleep deprivation 24 h before the assessment, no NSAR up to 14 days prior to the assessment, no otherwise antiaggregatory or immunomodulatory medication in the 14 days prior to the assessment. The participants were excluded if a psychiatric or somatic condition requiring medical attention was present. To ensure mental health our participants were screened for DSM-IV Axis I disorders through the German Version of the Mini International Neuropsychiatric Interview 5.0 M.I.N.I. (Sheehan et al. 1998). Thyroid replacement therapy was the only medication allowed at the time of study. All assessments were started between 8:00 and 8:30 am. Sociodemographic data were collected from all participants.

### Evaluation of Lifestyle Relevant Data

Physical activity was assessed using the self-administrable German version of the International Physical Activity Questionnaire (IPAQ) (Craig et al. 2003) and sleep quality was recorded with the German version of the Pittsburgh Sleep Quality Index (PSQI) (Buysse et al. 1989).

### Study Procedure

**Fig. 1:**
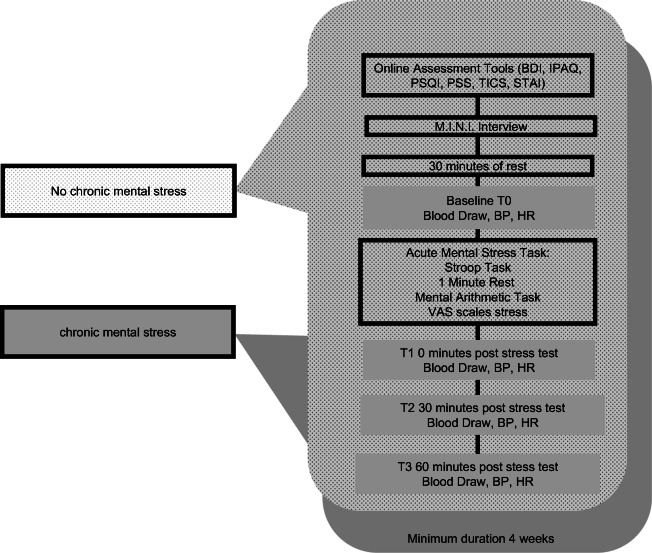
Study visit for each mental stress condition. Overview over the study design with the performed psychometric stress assessments, blood draws and physiological parameters

We assessed the participants at two study visits once in the absence of chronic mental stress and once in the presence of chronic mental stress. The order of the study conditions varied between the participants. The chronic mental stress condition (CS+) consisted of a major university exam with an average three-month preparation time. At each study visit a standardized acute mental stress (AS+) test was carried out. At each study visit blood was collected at four time points: after 30 min of rest (T0), immediately after the stress test (T1), 30 min post stress (T2) and 60 min post stress (T3). Blood pressure and heart rate were determined at each study visit from T0-T3. Figure 1 shows the detailed study protocol.

### Acute Stress Protocol

The acute stress test consisted of the STROOP color and word test and a standardized arithmetic test. The Stroop task widely used as a cognitive test is described to induce mental stress in laboratory settings by increasing cortisol levels, catecholamine levels and the heart rate (Teixeira et al. 2015; Wang et al. 2016; Usui and Nishida 2017). The mental arithmetic test is another standard laboratory stress test. Volunteers were instructed to engage in a serial mathematical tasks (Vella and Friedman 2009)**.** The Stroop task and the mental arithmetic task were both carried out for 2 × 2.5 min. The pressure on the participants was increased by (1) the examiner wearing a white coat and performing minimal interaction with the participants outside the study protocol, (2) a computer-generated metronome sound, (3) the introduction of social pressure via computer screen instruction reading, for example, “it is very important that you answer as many of the trials correctly as possible”, or “your score will be recorded and compared to the score of other individuals performing this test”. In the ‘Stroop task an additional stress factor was that participants could not perform the test at their own pace, but must adapt to the computer-generated pace, and in the mental arithmetic task stress was increased by the examiner pointing out wrong answers and asking to restart the calculation series after each wrong answer.

### Assessment of Stress-Related Psychometric Parameters

A screening for depressive symptoms was done with the “Becks’s Depression Inventory” (BDI) (Beck et al. 1961). The “State Trait Anxiety Inventory”(STAI) was used to assess anxiety symptoms (Laux et al. 1981). The “Perceived Stress Scale 14” (PSS-14) (Cohen et al. 1983), and the „Trier Inventory of Chronic Stress “(TICS) (Schulz and Schlotz 1999) were used for stress-related assessments. All are self-reporting instruments and digitalized versions were used. Individuals completed them in the days (maximum 7) prior to the study visit via an online link. On the assessment day parameters obtained online were checked with the participants and STAI state values were repeated. Additionally, the students had to rate their perceived amount of acute mental stress on a five-point Likert Scale following the acute mental stress test.

### Blood Sampling

A peripheral venous catheter was inserted into the antecubital vein of the non-dominant hand in all participants. Participants rested for 30 min following the insertion of the catheter before the first (resting) blood sample was drawn. Overall, 29.9 ml of venous blood were obtained at T0. For T1 to T3 an amount of 15.9 ml was collected. The first 2 ml were discarded during each draw to minimize preanalytical activation. Blood was drawn without stasis. The samples were collected in commercially available tubes (Sarstedt, Vienna, Austria). For FACS analysis blood was drawn into 2.9 ml Sarstedt CTAD containers. For aggregometry a 3.8 ml Citrate Buffer 9 NC/PFA vacutainer was used. For Cortisol analysis Li-heparin tubes were used. Cortisol was determined via Elecsys Cortisol II Test. Standard laboratory parameters including TSH and blood cell counts were determined in addition to the platelet parameters. The blood was processed immediately after collection.

### Measurement of Platelet Parameters

#### Determination of Clotting Time

The assessment of platelet aggregability was done as earlier described in Hüfner et al. (2015) using the PFA-100® (Siemens, Vienna, Austria) system.

#### FACS Analysis

**Fig. 2:**
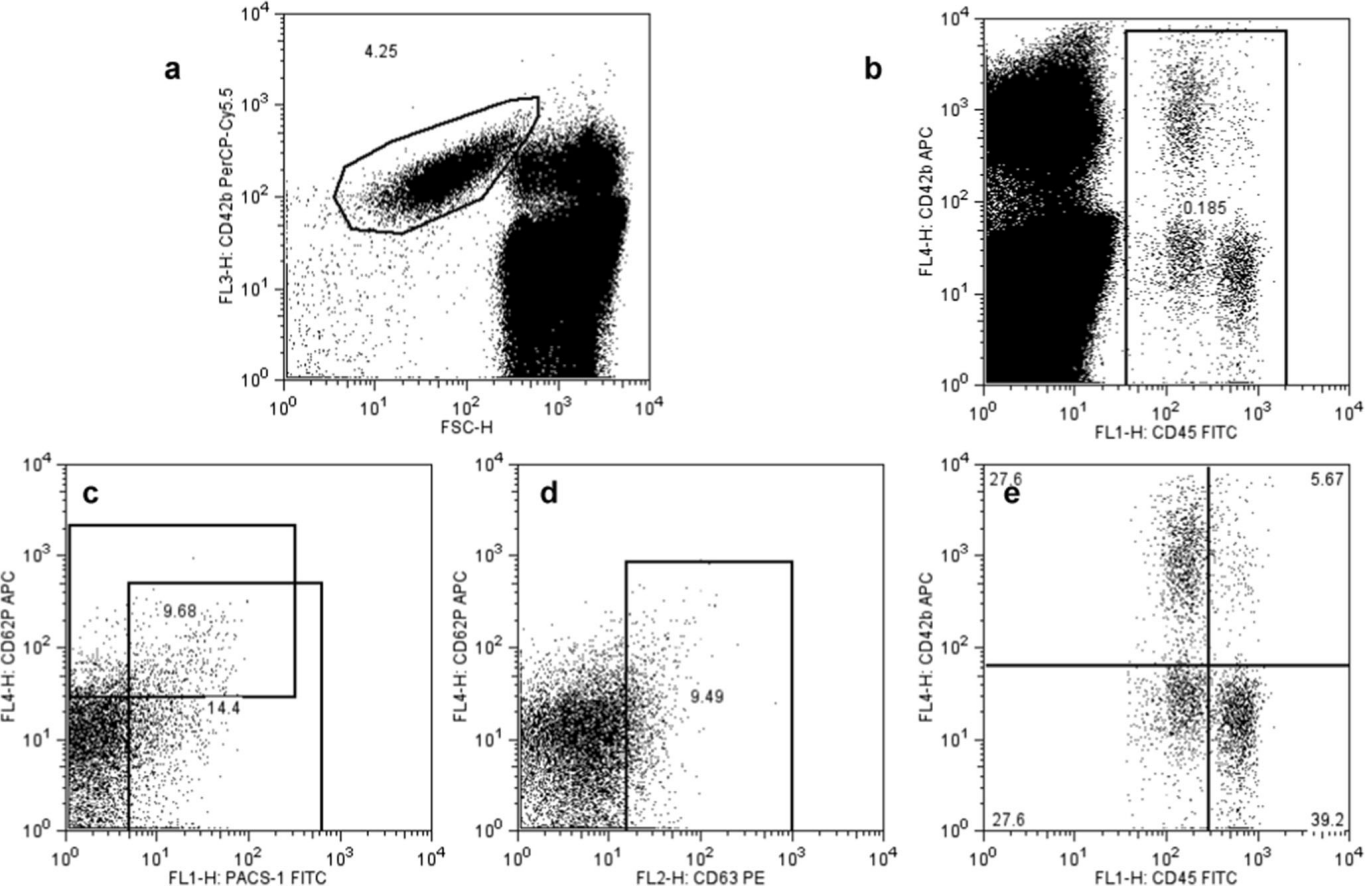
Example of platelet proinflammatory marker analysis by FACS. Platelets were defined by FSC (forward scatter) and CD42b (**a**), leukocytes based on CD45 expression (**b**). On platelets, expression of CD62b and PAC-1 (**c**) as well as CD63 (**d**) was determined. Platelet adhesion to leukocytes (**e**) was defined by CD42b positivity on CD45lo expressing granulocytes (upper left quadrant) or CD45hi monocytes and lymphocytes (upper right quadrant)

In order to determine the expression of activation markers on platelets, a mix of the following antibodies, PAC-1 FITC, CD63-PE (HSC6), CD62P-APC (AK-4), (all BD Biosciences) and CD42b-PerCP (HIP1, Biolegend), was added at pre-titrated concentrations to 100 μl CTAD blood in 5 ml polystyrene tubes (Falcon). Tubes were swiveled slightly and incubated for 10 min at room temperature in the dark. Thereafter, blood was diluted with 0.5 ml Phosphate buffered saline and fixed by the addition of another 0.5 ml ice cold paraformaldehyde. Samples were then immediately acquired on a FACSCalibur flow cytometer (BD Biosciences) and analyzed with FlowJo software (v8.87, Tree Star). An example of FACS analysis can be found in Fig. 2.

### Blood Pressure and Heart Rate

Blood pressure and heart rate were recorded following each blood draw i.e. at baseline and at 0, 30 and 60 min following the acute mental stress test using a manual blood pressure device and a stethoscope.

### Statistical Analysis

Sample characteristics are given as means and standard deviations. The results of lifestyle and stress-related psychometric assessments, FACS activation markers and aggregability as means and confidence intervals. Non-parametric analyses were performed to test for differences in lifestyle and stress-related psychometric parameters between the two study visits. Analysis of the impact of acute and chronic mental stress was based on a linear mixed model. Acute and chronic mental stress and their interaction term were included as main effects. To account for correlation between repeated assessments, we used a 1st order autoregressive covariance matrix and included an additional random intercept on patient level. Non-normally distributed variables were ln-transformed before inclusion in the model. Corrections for significant co-variates were performed were applicable. The platelet “recovery rate” was calculated as a separate analysis for comparison with the literature (Aschbacher et al. 2008). “Recovery” was conceptualized as a participant’s platelet ability to quickly recover, or return to a resting level of functioning, following a stressful event. It was calculated in the present study using (T1 and T3) and unadjusted values were compared between the CS+ and CS- condition using paired sample T-test. *P* < 0.05 was considered significant in these analyses. Analyses were done in SPSS 20.0 and 26.0.

## Results

### Participants

Fifty-five physically and mentally healthy volunteers were included in this study. All participants were male Caucasian medical students, mean age 23.1 ± 2.4 years; mean ± SD. A total number of seven reported allergies, three a history of asthma, one of migraine and four hypothyroidisms (no laboratory abnormalities at time of study). Important stress related psychometric data are given in Table 1. During chronic mental stress (CS+) overall physical activity was significantly reduced (Wilcoxon test, *p* = 0.007), as was the global sleep quality (Wilcoxon test, *p* = 0.008), although both parameters were still within the range of normal.

**Table 1:** Group characteristics and assessment days to exam, hours spent studying per day, sleep quality and physical activity

Parameter	Chronic Mental Stress (CS+)	No Chronic Mental Stress (CS-)	*p* value
Days to exam (days)	11.80 (10.14–13.46)	99.71 (85.82–113.60)	<0.001*
Hours spent studying/day outside lectures, seminars or other structured teaching activities (hours)	7.09 (6.50–7.68)	0.46 (0.04–0.89)	<0.001*
Sleep: PSQI global score (points)	4.14 (3.64–4.63)	3.36 (2.85–3.88)	0.008*
Physical activity: IPAQ overall MET minutes/weeks	3181.70 (2576.41–3786.99)	4175.33 (3356.39–4994.27)	0.007*
Physical activity: IPAQ moderate MET minutes/week	656.81 (399.49–914.14)	1045.65 (600.94–1490.35)	0.050*
Physical activity: IPAQ intense MET minutes/week	1617.25 (1193.33–2041.18)	2110.25 (1695.67–2524.84)	0.054

Results are given as means (95% confidence interval). Wilcoxon test. Significant p values are indicated by an asterisk

PSQI = Pittsburg Sleep Questionnaire Index (range 0–21 points; PSQI global score < 5:good sleep, PSQI global score > 5: disturbed sleep) (Buysse et al. 1989)

IPAQ = International physical activity questionnaire (WHO recommendations at least 600 MET minutes/week) (World Health Organization)

MET = metabolic equivalents

### Psychometric Measurements of Mental Stress

The CS+ condition lead to significant higher scores in the Perceived Stress Scale (PSS; Wilcoxon test, *p* = 0.001) and State-Anxiety Scale (STAI; Wilcoxon test, *p* < 0.001). In the Beck Depression Inventory (BDI) the participants rated significantly higher during CS+ (*p* = 0.045, Wilcoxon test). But in both stress conditions none of the participants showed any signs of depression. The TICS subscales „work overload“ (Wilcoxon test, *p* = 0.006), “discontentment with work“ (Wilcoxon test, *p* = 0.03), “chronic strain “ (Wilcoxon test, *p* = 0.018) and „chronic stress“ (Wilcoxon test, *p* = 0.016) were rating significantly higher in CS+. Following the acute mental stress tests (AS+) the participants rated acute subjective stress regarding the Stroop task higher in the CS+ condition compared to CS- (Wilcoxon test, *p* = 0.042) while no difference was found for the subjective stress due to the mental arithmetics between CS+ an CS-. For further details, see Table 2.

**Table 2:** Stress related psychometric parameters

Stress Parameter	Chronic Mental Stress (CS+)	No Chronic Mental Stress (CS-)	p Value
PSS (range 0–56 points)	18.37 (16.31–20.43)	15.16 (13.46–16.87)	0.001*
BDI (range 0–63 points)	3.13 (2.11–4.16)	2.24 (1.56–2.93)	0.045*
Trait anxiety scale (STAI, range 20–80 points)	47.19 (46.45–47.92)	47.19 (46.67–47.70)	0.966
State anxiety scale (STAI, range 20–80 points)	37.31 (34.64–39.99)	31.51 (29.55–33.47)	<0.001*
TICS work overload (range 0–32 points)	11.11 (9.45–12.77)	9.05 (7.67–10.42)	0.006*
TICS social overload (range 0–24 points)	7.00 (5.70–8.30)	7.33 (6.18–8.48)	0.571
TICS pressure to perform (range 0–36 points)	14.65 (12.81–16.50)	14.31 (12.72–15.90)	0.645
TICS discontentment with work (range 0–32 points)	10.24 (8.67–11.80)	8.82 (7.51–10.13)	0.030*
TICs excessive demands from work (range 0–24)	5.13 (4.07–6.19)	4.24 (3.43–5.04)	0.021*
TICS lack of social recognition (range 0–16 points)	3.56 (2.83–4.30)	3.47 (2.70–4.25)	0.778
TICS social tensions (range 0–24 points)	5.20 (4.25–6.15)	4.42 (3.53–5.31)	0.063
TICS social isolation (range 0–24 points)	6.45 (5.21–7.70)	5.45 (4.39–6.52)	0.114
TICS chronic worrying (range 0–16 points)	4.76 (3.87–5.66)	4.04 (3.32–4.75)	0.018*
TICS chronic stress (range 0–48 points)	12.69 (10.47–14.91)	10.64 (8.86–12.41)	0.016*
Stress from test Stroop (5 Point Likert scale)	3.33 (3.05–3.60)	3.13 (2.89–3.36))	0.042*
Stress from test arithmetics (5 Point Likert scale)	2.56 (2.30–2.81)	2.59 (2.29–2.89)	0.431

Stress-related psychometric parameters were collected once in the condition with chronic mental stress and once in the absence of chronic mental stress. Results are given as means of the scored points (95% confidence interval), Wilcoxon test was used, significant p values are indicated by an asterisk. Ranges of the respective tests or subtests are given to aid with interpretation

Abbreviations: PSS=Perceived Stress Scale

STAI = State Trait Anxiety Scale

BDI = Beck’s Depression Inventory

TICS = Trier Inventory of Chronic Stress

### The Response of Blood Pressure and Heart Rate in Response to Mental Stress

The effect of acute (AS+) and chronic (CS+) stress as well as their interactions on blood pressure and heart rate were analyzed using mixed linear model. Systolic, diastolic blood pressures as well as heart rate were all influenced by AS+ (*p* < 0.001, *p* = 0.006, p < 0.001 respectively) no influence of CS+ or interaction was found (Table 3 and 4).

**Table 3:** Results of laboratory and FACS analyses before (T0) and following an acute mental stress test (T1 to T3)

Parameter	T0	T1	T2	T3	P value
Heart rate per min	68.98 (67.26 -70.69)	69.92 (68.20 -71.64)	67.38 (65.67 -69.10)	66.10 (64.38 -67.82)	<0.001*
Diastolic blood pressure (mmHg)	76.72 (74.82–78.61)	78.01 (76.12–79.91)	76.19 (74.29–78.08)	75.50 (73.61–77.39)	0.006*
Systolic blood pressure (mmHg)	118.90 (116.93–120.86)	121.87 (119.90–123.83)	118.44 (116.48–120.41)	118.01 (116.04–119.97)	<0.001*
Platelets (G/l)	201.00 (191.18–210.81)	204.97 (195.14–214.80)	202.86 (193.03–212.69)	204.25 (194.44–214.07)	0.020*
Leucocytes (G/l)	5.40 (5.16–5.65)	5.57 (5.33–5.82)	5.25 (5.01–5.50)	5.41 (5.17–5.65)	<0.001*
Cortisol (μg/l)	169.41 (160.16–178.66)	155.66 (146.40–164.93)	125.80 (116.54–135.07)	113.43 (104.22–122.63)	<0.001*^2^
CD62P+ (%)	6.25 (5.50–7.00)	5.96 (5.21–6.71)	5.31 (4.56–6.06)	4.76 (4.02–5.51)	<0.001*^1^
CD63+ (%)	3.74 (3.17–4.30)	3.78 (3.21–4.35)	3.47 (2.91–4.04)	3.25 (2.69–3.82)	0.011*^1,2^
PAC-1+ (%)	9.82 (8.52–11.12)	9.68 (8.38–10.98)	8.95 (7.66–10.25)	7.94 (6.65–9.23)	<0.001*^2^
CD45high42+ (PLA) (%)	5.97 (5.30–6.64)	6.01 (5.34–6.69)	5.59 (4.92–6.27)	5.40 (4.73–6.08)	0.019*^2^
Col/ADP (sec)	88.67 (85.58–91.77)	86.34 (83.23–89.46)	90.11 (86.99–93.23)	87.51 (84.41–90.62)	0.020*^1^
Col/EPI (sec)	127.77 (120.19–135.35)	131.89 (124.28–139.50)	134.53 (126.91–142.15)	129.71 (122.14–137.29)	0.084^1^

Results are presented as estimated marginal means and are given as mean (95% confidence interval) for *n* = 55 participants. A linear mixed model including acute and chronic mental stress and significant co-variables were applicable was used for analysis. Data on the effect of chronic mental stress obtained using the same model for analysis are presented in Table 4. Statistically significant *p* values are indicated by an asterisk. The interaction of acute and chronic stress was not significant in any of the analyses and the p values are thus not reported separately. ^1^adjusted for Pittsburgh sleep quality index (PSQI), ^2^adjusted for International physical activity questionnaire (IPAQ), ^3^adjusted for order of examinations (CS+ or CS- first)

T0 = baseline after 30 min of rest

T1 = immediately after acute mental stress test

T2 = 30 min after acute mental stress test

T3 = 60 min after acute mental stress test

PLA = platelet leucocyte aggregates

col/ADP: aggregability measured with PFA using ADP

col/EPI: aggregability measured with PFA using epinephrine

**Table 4:** Results of laboratory and FACS analyses on the effect of chronic mental stress

Parameter	Chronic Stress (CS+)	No Chronic Stress (CS-)	P Value
Heart rate per min	68.68 (66.89–70.48)	67.51 (65.73–69.29)	0.254
Diastolic blood pressure (mmHg)	77.45 (75.45–79.44)	75.76 (73.79–77.73)	0.115
Systolic blood pressure (mmHg)	120.03 (117.96–122.10)	118.58 (116.54–120.62)	0.196
Platelets (G/l)	200.00 (189.95–210.05)	206.54 (196.53–216.55)	0.054
Leucocytes G/l	5.47 (5.21–5.72)	5.36 (5.11–5.62)	0.309
Cortisol (μg/l)	140.07 (130.23–149.91)	142.08 (132.41–151.75)	0.518^2^
CD62P+ (%)	5.48 (4.69–6.27)	5.66 (4.88–6.43)	0.566^1^
CD63+ (%)	3.86 (3.26–4.47)	3.26 (2.66–3.85)	0.009*^1,2^
PAC-1+ (%)	8.72 (7.34–10.11)	9.47 (8.11–10.83)	0.356^2^
CD45high42b + (PLA) (%)	5.73 (5.00–6.45)	5.76 (5.05–6.48)	0.429^2^
Col/ADP (sec)	89.23 (86.02–92.44)	87.09 (83.96–90.22)	0.317^1^
Col/EPI (sec)	136.12 (128.05–144.19)	125.83 (117.93–133.73)	0.031*^1^

Results are presented as estimated marginal means and are given as mean (95% confidence interval) for n = 55 participants. A linear mixed model including acute and chronic mental stress and significant co-variables were applicable was used for analysis. Data on the effect of acute mental stress obtained using the same model for analysis are presented in Table 3. Statistically significant p values are indicated by an asterisk. The interaction of acute and chronic stress was not significant in any of the analyses and the p values are thus not reported separately. ^1^adjusted for Pittsburgh sleep quality index (PSQI), ^2^adjusted for International physical activity questionnaire (IPAQ), ^3^adjusted for order of examinations (CS+ or CS- first)

col/ADP: aggregability measured with PFA using ADP

col/EPI: aggregability measured with PFA using epinephrine

### Effects of Acute Mental Stress on Platelet Proinflammatory Markers and Platelet Aggregability

**Fig. 3:**
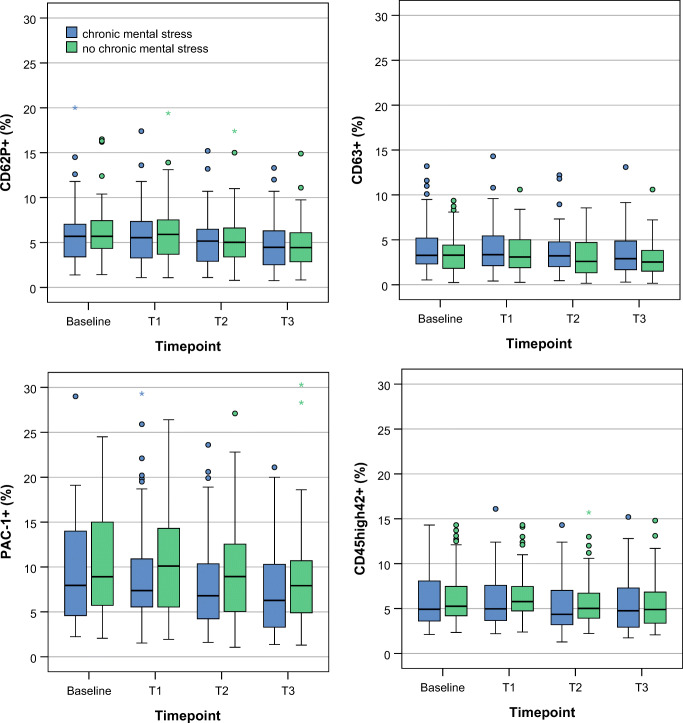
Boxplots of observed values for proinflammatory platelet parameters as determined by FACS analysis. Boxplots represent medians of the observed values with interquartil ranges. *N* = 55. p values from the mixed linear model can be found in Tables 3 and 4. T0 = baseline after 30 min of rest. T1 = immediately after acute mental stress test. T2 = 30 min after acute mental stress test. T3 = 60 min after acute mental stress test.

**Fig. 4:**
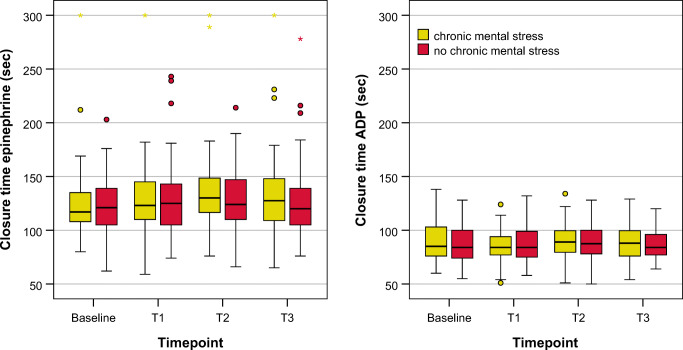
Boxplots of observed values for PFA closure times. Boxplots represent medians of the observed values with interquartil ranges. N = 55. *p*-values from the mixed linear model can be found in Tables 3 and 4. T0 = baseline after 30 min of rest. T1 = immediately after acute mental stress test. T2 = 30 min after acute mental stress test. T3 = 60 min after acute mental stress test.

A mixed linear model including acute (AS+) and chronic stress (CS+) as well as their interactions was used for analysis. The acute mental stress task influenced the proinflammatory marker set significantly: the platelet markers CD62P (*p* < 0.001), CD63 (*p* = 0.011), PAC-1 (p < 0.001) and the number of platelet leucocytes aggregates (CD45highCD42b+, PLA; *p* = 0.019) were significantly influenced by the factor AS+. Of note, %CD 62P+ platelets and % PAC-1+ platelets were highest at T0 and then decreased gradually until T3. The number of platelets (*p* = 0.020) and leucocytes (p < 0.001) as well as plasma cortisol (p < 0.001) were also significantly influenced by AS+. Cortisol showed the highest levels at T0 and the lowest at T3. AS+ lead to a significant change in platelet aggregability in response to ADP (p = 0.020), whereas the response to epinephrine was not significantly altered. The interaction of acute and chronic stress was not significant in any of the analyses. Means and CI of each marker can be found in Table 3, raw data are presented in Figs. 3 and 4.

### Influence of Chronic Mental Stress on FACS Proinflammatory Platelet Parameters and Platelet Aggregability

Chronic mental stress significantly increased the proinflammatory marker CD63 on platelets (*p* = 0.009). The other proinflammatory platelet markers CD62P (*p* = 0.566), PAC-1 (*p* = 0.356) and PLA formation (CD42b + CD45high; *p* = 0.429) were not significantly influenced by the factor CS+. Platelet counts were decreased in CS+ (*p* = 0.054). Plasma cortisol levels (*p* = 0.518) were not significantly influenced by the factor CS+. No interaction between the chronic mental stress and the acute mental stress condition could be found for any of the analyses. The aggregability using PFA due to epinephrine was significantly influenced by the factor CS+ (*p* = 0.031) with CS+ leading to prolonged closure times, while response to ADP was not affected (*p* = 0.317). Means and CI of each marker can be extracted from Table 4, raw data are presented in Figs. 3 and 4.

### Recovery of Platelet Parameters in the Condition with and without Chronic Mental Stress

We performed an additional analysis of platelet function recovery for the condition CS+ and CS- outside the mixed linear model reported above. Observed values were compared by analyzing the difference between T1 and T3 in the condition CS+ and CS-. Platelet CD62P showed impaired recovery i.e. a prolonged activation in the CS+ condition (CD62P+ (%) CS+: mean 0.85 (95% CI 0.45–1.25); CS-: mean 1.52 (95% CI 1.06–1.98), T-Test, *p* = 0.023). The recovery of the other platelet activation markers CD63 (T-Test, *p* = 0.580), PAC-1 (T-Test, *p* = 0.579) and CD45highCD42b + (T-Test, *p* = 0.121) was not significantly altered in CS+ condition compared to CS-.

## Discussion

In the present study we assess the effect of acute and chronic mental stress as well as a combination of both on platelet proinflammatory markers (CD62P, CD63, PAC-1 and PLA) as well as on platelet aggregability. The most important findings of our study are: 1) AS+ significantly influences the platelet proinflammatory markers CD62P+, CD63+, PAC-1+ and PLA as well as cortisol. 2) CS+ affects platelet expression of CD63, 3) No interaction of CS+ and AS+ were found when measuring platelet activation or aggregability 4) A significantly impaired recovery of CD62P over 60 min was found following acute mental stress exposure during CS+ compared to CS-. This points towards a prolonged proinflammatory acute stress response in the CS+ condition.

### Acute Mental Stress Increases Platelet Proinflammatory Activity

The effect of acute mental stress on platelet reactivity has been extensively investigated in different populations such as cardiovascular patients, depressed patients and healthy individuals using variable biomarkers. This heterogeneity in the studies in combination with divergent analytical methods to measure platelet activation have led to conflicting results (Camacho and Dimsdale 2000; Malkoff et al. 1993; Wallén et al. 1997; Brydon et al. 2006). In case studies assess the soluble and not platelet bound proinflammatory markers such as CD62P additional heterogeneity arises from the fact that this is not solely derived from platelets but also from endothelial cells (Blann et al. 2003). Thus FACS analysis of surface bound proinflammatory activation markers and PLA are now considered a more accurate tool. PLA monitoring in particular seems to be a very useful instrument because the PLA are stable whereas surface bound CD62 is cleaved after some time (Michelson et al. 2001). FACS whole blood analysis techniques are less prone to preanalytic activation (Pearson et al. 2009; Juster et al. 2010).

Acute mental stress has been shown to lead to an increase in several platelet proinflammatory markers, here are some examples: beta thromboglobulin was increased in healthy volunteers and stable patients following myocardial infarction (Markovitz et al. 1996) as well as in hypertonic patients (Tomoda et al. 1999). Healthy controls and patients with coronary artery disease showed a significant increase in PLA following mental stress with peak levels 30 min post stress (Strike et al. 2004). Furthermore in coronary artery disease patients an acute mental stress test lead to a significant increase in a proinflammatory platelet marker set among them CD62P surface expression, % CD62P+ and % mononuclear cell platelet aggregates (Hill and Butler 1991; Reid et al. 2009). The effect of mental stress on platelet proinflammatory markers has also been observed in elderly individuals in a longitudinal study (Aschbacher et al. 2009b). Similar to these findings acute mental stress in the present study elicited a significant change in the proinflammatory markers CD62P+, CD63+, PAC-1+ and PLA. Physiological parameters such as heart rate and blood pressure followed the same activation pattern as the platelet pro-inflammatory marker set. For the markers CD62P+, PAC-1 and also for cortisol we recorded the highest levels even before the acute stress task. The elevated baseline levels at T0 in our study may be attributed to an anticipatory effect, regarding cortisol such an effect has been described (Engert et al. 2013). After activation, P-Selectin is mobilized to the external plasma membrane within minutes. This increase in P-Selectin expression is transient (Mantovani and Dejana 1998). Additionally it is possible that the decrease in CD62P+ platelets observed at 1 h post acute mental stress could be related to the release of microvesicles. Microvesicles are released from platelets upon activation and retain many aspects and markers of their “parents” such as immunomodulatory function (Vajen et al. 2015).

### Chronic Mental Stress Affects Platelet CD63 Expression

In our assessment the chronic mental stress condition solely had a significant effect on the increase of platelet marker CD63. This may be explained by the fact that our study design (major university exam) did not result in high enough chronic stress load (see limitation section). The amount of perceived stress seems to be a key player in the stress response system, the students in the current study rated relatively low amounts of perceived stress although a significant increase could be observed in the chronic mental stress condition compared to the condition without chronic mental stress. This increase was within the values reported for university students in challenging situation (Marshall et al. 2008). Furthermore our participants were sleeping well and still performing within the recommended range of physical activity (Craig et al. 2003; Hagovska et al. 2018). In other studies chronic mental stress in the form of caregiving has been shown to be associated with increased CD62P reactivity (Aschbacher et al. 2008). While this demonstrates that chronic mental stress due to an exam preparation certainly differs from that of caregiving or childhood trauma, it has nevertheless been shown previously to be associated with immunological and other biological changes (Marshall et al. 1998; Peters et al. 2017).

### Impaired CD62P Recovery Following Chronic Mental Stress: An Other Useful Tool in Assessing Allostatic Load?

In the present study we found a prolonged proinflammatory platelet response (delayed recovery) for CD62P in the chronic mental stress condition. Prolonged circulation of PLA and expression of surface bound platelet activation markers following acute mental stress has been found in patients with coronary artery disease (Strike et al. 2004) and chronic mental stress due to caregiving (Aschbacher et al. 2008, a, b). In the sense of allostasis, a prolonged proinflammatory platelet response during chronic mental stress suggests that the adaptive process which should tackle adverse situations, is not terminated promptly after the acute mental stress stimulus has subsided. The gathered evidence proposes that the impaired recovery of the proinflammatory marker CD62P may be used as a very sensitive tool in evaluating the stress sensitizing effect of chronic mental stress. The impaired recovery of CD62P in our sample may be an early sign of the “wear and tear” (Strain 2018) of allostatic processes. For further directions, it should be investigated if impaired CD62P recovery could be a first sign of chronic stress related “allostatic load”. Stress hormones in conjunction with pro- and anti-inflammatory cytokines such as IL-6 have also been proposed to represent biomarkers of the allosteric load (McEwen 2005; Juster et al. 2010).

### The Effect of Different Mental Stress Conditions on Platelet Aggregability

The acute mental stress task significantly influenced the aggregation measured by PFA due to col/ADP whereas col/EPI was not significantly changed. The chronic mental stress condition lead to a significant prolongation of col/EPI only. Our findings are partly contrasting previous research where no effect of chronic mental stress on PFA testing was found (Hüfner et al. 2015). In line with our present findings it has been shown in healthy participants that the Stroop task doesn’t lead to any aggregability changes during the test but to decreased aggregability in the aftermath (Naesh et al. 1993). Similarly (Wallén et al. 1997) were able to detect an increased platelet aggregability during the Stroop task in cardiovascular disease patient, but in healthy volunteers a tendency of the opposite effect. Therefor it may be assumed that that healthy individuals are less prone to acute stress task induced hypercoagulability.

## Limitations

The chronic mental stress paradigm presented in this study was not strong or enduring enough to evoke reactions in all of the proinflammatory platelet markers assessed and possibly had a more positive subjective connotation to the participants as e.g. caregiving or childhood trauma stress. Although we were able to detect a significant influence of the chronic mental stress condition on several psychometric parameters like BDI, PSS, the TICS subscales and the State Anxiety Inventory, our sample did not show any core symptoms of persistent chronic mental stress like depressive symptoms, sleeping problems or reduced physical activity. Due to the homogeneity of our sample during the analysis we were not able to stratify according to different psychometric properties. Some of the proinflammatory platelet markers were elevated before the onset of the acute mental stress task, and then returned to lower values during recovery. We suppose an anticipatory effect, but our study was not designed to evaluate such an effect.

## Conclusion

According to our present finding our platelet proinflammatory marker set seems to be a feasible tool in assessing the differential effects of acute and chronic mental stress. The proinflammatory platelet response induced by mental stress helps to explain the link between mental stress and somatic, especially cardiovascular disease. This is especially true for the prolonged activation of platelets in response to acute stress which was observed in the chronic stress condition. Further investigations are needed to characterize the stress response in different groups according to their levels of perceived stress, to their level of depressed symptoms and the most importantly childhood adversities should be taken into account. It is also of great importance to include women as they might to display different psychometric properties regarding stress responsiveness.
